# Vaginal *Lactobacillus* spp. Dominance in Late Pregnancy and Neonatal Anthropometric Outcomes: A Prospective Observational Study

**DOI:** 10.3390/jcm15134867

**Published:** 2026-06-23

**Authors:** Oana Liliana Atomei, Andrei Lobiuc, Petronela Vicoveanu, Maricela Cobuz, Monica Tarcea

**Affiliations:** 1 Doctoral School, G.E. Palade University of Medicine, Pharmacy, Science, and Technology of Targu Mures, 540139 Targu Mures, Romania; 2 Department of Biological and Morphofunctional Sciences, Faculty of Medicine and Biological Sciences, Stefan cel Mare University of Suceava, 720229 Suceava, Romania; andrei.lobiuc@usm.ro; 3 Department of Medical-Surgical and Complementary Sciences, Faculty of Medicine and Biological Sciences, Stefan cel Mare University of Suceava, 720229 Suceava, Romania; petronela.vicoveanu@usm.ro (P.V.); maricela.cobuz@usm.ro (M.C.); 4 Department of Obstetrics and Gynecology, Emergency Clinical County Hospital Sfantul Ioan cel Nou, 720224 Suceava, Romania; 5 Department of Neonatology, Emergency Clinical County Hospital Sfantul Ioan cel Nou, 720224 Suceava, Romania; 6 Department of Community Nutrition and Food Safety, G.E. Palade University of Medicine, Pharmacy, Science, and Technology of Targu Mures, 540139 Targu Mures, Romania; monica.tarcea@umfst.ro

**Keywords:** vaginal microbiota, *Lactobacillus*, pregnancy, fetal growth, birth weight, head circumference

## Abstract

**Background/Objectives**: Vaginal microbiota is relevant to pregnancy, but evidence on neonatal anthropometry is mainly molecular and focused on pre-term birth. This study evaluated whether microscopy-based vaginal lactobacillary dominance in late pregnancy is associated with neonatal anthropometric measures after accounting for maternal and gestational determinants. **Methods**: This prospective observational study included 144 mother–newborn pairs recruited at a tertiary hospital in Romania between February and August 2025. Gram-stained smears were assessed for lactobacillary dominance, leukocyte density, and candidiasis; a composite vaginal indicator was derived. Outcomes were birth weight, length, and head circumference. Associations were assessed using correlation, adjusted regression, hierarchical models, and sensitivity analyses. **Results**: Lactobacillary dominance was not associated with birth weight or length in bivariate analyses, but correlated weakly with head circumference (ρ = 0.186, *p* = 0.025). In adjusted models, it was nominally associated with higher birth weight (B = 133.5 g, *p* = 0.043) and larger head circumference (B = 0.47 cm, *p* = 0.034), but not birth length; these associations did not remain significant after multiple-testing correction. Incremental explanatory contribution was modest (ΔR^2^ = 0.022 and 0.025), and associations attenuated after socioeconomic adjustment. Leukocyte density and candidiasis were not associated with outcomes; intermediate versus balanced vaginal status was associated with lower birth weight and head circumference in exploratory analyses. **Conclusions**: Routine microscopy-based lactobacillary dominance showed modest nominal associations with birth weight and head circumference, limited incremental explanatory value, and no robustness after multiplicity correction. These findings suggest a context-dependent association with limited clinical relevance.

## 1. Introduction

The vaginal microbiota is increasingly recognized as part of the biological environment during pregnancy, with potential implications for maternal health, pregnancy progression, and neonatal outcomes. In an uncomplicated pregnancy, the vaginal ecosystem is typically characterized by low diversity and relative stability, with dominance of *Lactobacillus* spp., a pattern associated with maintenance of low vaginal pH and restriction of potentially pathogenic anaerobes [1,2]. Most microbiome-based studies in pregnancy have focused on adverse obstetric outcomes, particularly preterm birth, and have relied largely on molecular approaches such as 16S rRNA sequencing to characterize vaginal microbial communities [2,3,4,5].

Across systematic reviews and cohort studies, reduced *Lactobacillus* dominance and dysbiotic community profiles have generally been associated with higher risk of adverse pregnancy outcomes, although findings remain heterogeneous across populations, ethnic groups, sampling frames, and analytic approaches [2,3,4,5]. Most of this evidence is derived from sequencing-based studies that use community state type (CST) frameworks and primarily address gestational endpoints rather than neonatal anthropometric outcomes such as birth weight or length [3,4,5,6]. For example, large cohort studies using 16S rRNA gene sequencing and CST classification primarily evaluate associations with spontaneous preterm birth, with limited assessment of neonatal anthropometric variability [4,6].

In contrast, clinically accessible microscopic markers of the vaginal ecosystem have been less frequently examined in relation to neonatal outcomes. In routine obstetric settings, direct molecular profiling is often unavailable, whereas simple microscopy-based parameters such as lactobacillary dominance, leukocyte density, and Gram-stain-derived measures remain feasible and inexpensive. However, evidence linking these microscopy-based markers to neonatal anthropometric measures remains limited, highlighting a gap between research-intensive microbiome profiling and clinically applicable assessment tools [3,5,6]. Pregnancy-related microbiota studies indicate that microbial composition varies across the perinatal period and is influenced by multiple maternal and clinical factors, including timing of sampling, maternal body mass index (BMI), and antibiotic exposure [7], further complicating the interpretation of simplified microscopy-based markers.

There is a biological rationale for focusing specifically on vaginal lactobacillary dominance. *Lactobacillus*-dominated communities are generally considered protective because they support an acidic vaginal microenvironment, restrict overgrowth of anaerobic and potentially pathogenic taxa, and contribute to epithelial and immune homeostasis [5,8]. Experimental and translational work suggests that vaginal epithelial function is linked to the local microbiome, metabolome, and inflammatory signaling pathways, indicating interactions between microbial balance and host mucosal responses [9].

In contrast, depletion of lactobacilli has been associated with greater microbial diversity, altered host–microbe interactions, and less favorable pregnancy-related profiles [4,5]. Notably, meta-analytic evidence indicates that low vaginal *Lactobacillus* dominance is associated with increased risk of preterm birth, whereas *Lactobacillus crispatus* dominance appears to be the most favorable profile [3,5]. These associations relate primarily to gestational outcomes, and their extension to fetal growth and neonatal anthropometry remains insufficiently characterized.

At the same time, it remains unclear whether vaginal lactobacillary dominance remains associated with neonatal size after accounting for established determinants of fetal growth, or whether it primarily reflects these broader maternal and gestational factors. Gestational age is the primary determinant of birth anthropometry, and maternal factors such as smoking, adiposity, and age are also relevant to fetal growth trajectories [10,11,12]. In addition, evidence from observational studies suggests that socioeconomic status, dietary patterns, and lifestyle factors during pregnancy are associated with both preterm birth and neonatal anthropometric outcomes, further highlighting the multifactorial determinants of fetal growth [13]. Tobacco exposure has been associated with perinatal microbiome alterations [14].

These factors may also be associated with variation in the maternal microbiome and inflammatory milieu, raising the possibility that observed associations between vaginal microbiota and neonatal outcomes could be confounded or mediated by these pathways. However, whether clinically assessed lactobacillary dominance adds information beyond these established determinants remains uncertain.

Another consideration is that microscopic vaginal findings may capture multiple biological dimensions beyond bacterial composition alone. Increased leukocyte density on vaginal smear is widely regarded as an indirect marker of local inflammatory activation in pregnancy, reflecting host immune responses to microbial imbalance or infection [15,16], while vaginal *Candida* colonization is frequently encountered during pregnancy and reflects additional host–microbe interactions within the vaginal ecosystem [17,18]. Nevertheless, these markers may not reflect the same biological processes as lactobacillary dominance, and their relative contribution to neonatal anthropometric variation remains uncertain. Given these considerations, the present study focuses on a simplified, clinically accessible marker of vaginal ecological status—lactobacillary dominance—rather than detailed microbial profiling.

This study aimed to evaluate whether vaginal lactobacillary dominance, assessed by routine microscopy in late pregnancy, is associated with neonatal anthropometric outcomes at birth and whether this association persists after accounting for gestational age and key maternal factors. Secondary objectives were exploratory and included descriptive comparisons, bivariate analyses, and assessment of the incremental contribution of lactobacillary dominance in multivariable models.

## 2. Materials and Methods

### 2.1. Study Design, Setting, and Timeframe

This hospital-based prospective observational cohort study used a single antenatal assessment of vaginal microscopic status and maternal exposures during the third trimester of pregnancy, followed by ascertainment of neonatal outcomes from clinical records. The temporal sequence of data collection allowed vaginal microscopic markers and maternal covariates to be measured before the neonatal outcomes occurred.

The study was conducted at the Department of Obstetrics and Gynecology and the Department of Neonatology of the Emergency County Clinical Hospital Sfantul Ioan cel Nou Suceava, Romania, a regional hospital with a neonatal intensive care unit and rooming-in care. Recruitment took place during routine prenatal obstetric consultations between February and August 2025, and final neonatal data were obtained from the medical records and the hospital electronic system of the same institution after delivery.

### 2.2. Participants and Sampling

Eligible participants were pregnant women aged ≥ 18 years with a singleton live fetus. Recruitment was consecutive during the predefined study period. A total of 147 women were initially enrolled in the third trimester; three were excluded due to missing outcome data (delivery in other hospitals), resulting in a final sample of 144 mother–newborn pairs.

Exclusion criteria were multiple pregnancy, neonatal macrosomia, defined as birth weight ≥4000 g, maternal diabetes, fetal, neonatal, or maternal death, major obstetric complications expected to substantially alter pregnancy course or perinatal management (for example, severe preeclampsia or placenta previa with active bleeding), severe uncontrolled chronic disease, and systemic corticosteroid or antibiotic treatment in the preceding 30 days. Given the sample size, the study was not specifically powered to detect small effect sizes.

### 2.3. Data Collection and Measures

Data were collected in a temporally structured manner. During the third trimester of pregnancy, participants completed a questionnaire prior to vaginal sample collection. The questionnaire provided maternal variables, including maternal age, pregestational weight and height (used to derive pregestational BMI), smoking during pregnancy, educational level, and household income. Obstetric and neonatal data were subsequently extracted from standard maternal and neonatal medical records after delivery. These included gestational age at delivery, parity, neonatal anthropometric measures (birth weight, birth length, and head circumference), and newborn sex.

Pregestational BMI was used to characterize maternal weight status and was classified according to World Health Organization criteria (<18.5 kg/m^2^ underweight, 18.5–24.9 kg/m^2^ normal weight, 25.0–29.9 kg/m^2^ overweight, and ≥30.0 kg/m^2^ obesity) [19].

### 2.4. Vaginal Microscopic Assessment

Vaginal samples were collected during prenatal consultations using a sterile speculum. Secretions were obtained from the mid-vaginal wall, smeared on glass slides, Gram-stained, and analyzed in a clinical laboratory operating within the hospital according to routine diagnostic protocols for vaginal smear microscopy. Samples were transported at room temperature or at +4 °C and were processed and reported within the timeframe specified in the laboratory protocol. Microscopic examination was performed within the routine microbiology laboratory workflow. According to the laboratory’s internal quality-control procedure, smears were evaluated within a two-specialist workflow. During the study period, smears were read by different designated staff members, and the results were checked and validated by a laboratory physician. Internal quality-control procedures also included periodic independent double reading of samples to monitor result concordance. At the time of microscopic assessment, neonatal outcomes were not yet available because delivery occurred after antenatal sampling; therefore, slide assessment was not informed by birth weight, birth length, or head circumference. The Nugent score was reported as part of this routine microscopic assessment; given the predominance of normal flora and the absence of bacterial vaginosis cases, it was not included in inferential analyses. Vaginal lactobacillary dominance was defined as the presence of >30 lactobacilli per microscopic field, a semiquantitative microscopy-based threshold used to identify smears with high lactobacillary density and clear *Lactobacillus* predominance, in line with established microscopic approaches to vaginal flora assessment [8,16]. This threshold was used as an operational marker of lactobacillary predominance under routine Gram-stained microscopy and should not be interpreted as equivalent to molecularly defined *Lactobacillus*-dominant community state types or as a species-level microbiome definition. Vaginal leukocyte density was dichotomized as frequent/numerous versus rare/relatively frequent. Microscopic candidiasis was defined as the presence of yeast forms and/or hyphae/pseudohyphae on microscopy and coded as present/absent. A composite vaginal microscopic indicator was constructed based on lactobacillary dominance and leukocyte density to provide a pragmatic representation of vaginal ecological status. This approach was adopted to retain biologically meaningful variability despite limited Nugent score dispersion. Three ordinal categories were defined: balanced (lactobacillary dominance present and low leukocyte density), intermediate (all other combinations), and unbalanced (absence of lactobacillary dominance and high leukocyte density).

### 2.5. Outcome Variables

Neonatal anthropometric outcomes included birth weight (g), head circumference (cm), and birth length (cm). These three variables were defined as the primary neonatal anthropometric outcome for the family because they are standard complementary indicators of neonatal size at birth [20]. Preterm birth (<37 weeks of gestation) was not used as the dependent variable in the inferential models because the microbiology subsample included only 7 preterm births.

### 2.6. Main Exposure and Covariates

The main exposure was vaginal lactobacillary dominance. Based on biological plausibility and sample size constraints, adjusted models included a parsimonious set of covariates: pregestational BMI (continuous), smoking during pregnancy (yes/no), gestational age at delivery (weeks), and newborn sex (male/female). These variables were selected for the primary model because they are established determinants of neonatal anthropometric measures [10,11,12,13,14,20] and were considered the minimum core adjustment set needed to account for major maternal, gestational, and neonatal influences while avoiding overfitting in a modest sample. Maternal age and parity were not included in the primary model to preserve parsimony, but were evaluated in sensitivity analyses to examine whether their inclusion materially changed the association estimates. Additional extended models included maternal education and household income. These socioeconomic variables were treated as extended covariates because they may represent upstream determinants of both vaginal ecological status and neonatal growth; therefore, adjustment for them was used to evaluate potential residual confounding, shared upstream pathways, and model-specification dependence. The attenuation of the associations after socioeconomic adjustment was considered central to interpretation rather than confirmatory of an independent biological effect. Residual confounding due to unmeasured or imprecisely measured variables cannot be excluded.

### 2.7. Statistical Analysis

Continuous variables were summarized using means and standard deviations or medians and interquartile ranges, as appropriate to their distribution; categorical variables were summarized as counts and percentages. Maternal, neonatal, and microscopic characteristics were compared according to vaginal lactobacillary dominance using independent-samples *t* tests or Mann–Whitney U tests for continuous variables and chi-square tests or Fisher’s exact tests for categorical variables, as appropriate.

The primary hypothesis was that vaginal lactobacillary dominance in late pregnancy is associated with neonatal anthropometric status at birth, as represented by the primary anthropometric outcome family defined above, after accounting for key maternal and gestational determinants.

Bivariate associations between vaginal lactobacillary dominance and neonatal anthropometric variables were examined using Spearman’s rank correlation coefficient. Given the binary nature of vaginal lactobacillary dominance, these correlations were interpreted as nonparametric measures of association that reflect group differences rather than linear relationships. Separate linear regression models were then fitted for each neonatal anthropometric outcome. For each outcome, an unadjusted model that included only vaginal lactobacillary dominance was first estimated, followed by an adjusted model including the prespecified covariates. Standardized regression coefficients were additionally reported for lactobacillary dominance in the adjusted models to facilitate comparison of relative effect sizes across neonatal anthropometric outcomes.

To evaluate whether lactobacillary dominance provided information beyond major maternal and gestational determinants, hierarchical linear regression models were additionally fitted for each neonatal anthropometric outcome. Variables were entered in three conceptually ordered blocks: (1) maternal factors (pregestational BMI and smoking during pregnancy); (2) gestational and neonatal factors (gestational age at delivery and newborn sex); and (3) vaginal lactobacillary dominance.

Model diagnostics were assessed for the adjusted linear regression models by examining standardized residuals, residual-versus-predicted plots, normal probability plots, Cook’s distance, leverage values, and collinearity statistics, including tolerance and variance inflation factors.

For these models, R^2^ and adjusted R^2^ were reported for each block, along with the ΔR^2^ attributable to the addition of the final block. This approach was used to quantify the incremental explanatory contribution of vaginal lactobacillary dominance beyond conventional determinants of neonatal anthropometric variation.

Sensitivity analyses were conducted by additionally entering maternal age and parity into the adjusted models. All tests were two-sided, and statistical significance was defined as *p* < 0.05. To address multiplicity across the three primary neonatal anthropometric outcomes, a sensitivity analysis was performed using Bonferroni correction and Benjamini–Hochberg false discovery rate correction for the *p*-values corresponding to lactobacillary dominance in the adjusted models. Data management was performed in Microsoft Excel, version 2108, included in Microsoft Office LTSC Standard 2021 (Microsoft Corporation, Redmond, WA, USA), and statistical analyses were conducted in IBM SPSS Statistics, version 26 (IBM Corp., Armonk, NY, USA).

A formal a priori sample size calculation was not performed, as the study was based on a consecutive clinical sample recruited during a predefined time period. The primary analyses and the core adjustment set were defined a priori based on biological plausibility and prior literature. Extended models including socioeconomic variables (maternal education and household income) were conducted to assess robustness, model-specification dependence, and potential residual confounding. Analyses involving additional vaginal microscopic markers (vaginal leukocyte density, microscopic candidiasis, and the composite vaginal microscopic indicator) and additional sensitivity models were considered secondary or exploratory.

### 2.8. Ethical Considerations

The study was conducted in accordance with the Declaration of Helsinki and received approval from the relevant institutional ethics committees (Approval Nos. 29/20.06.2024 and 3295/08.07.2024). All participants provided written informed consent prior to participation. Data were anonymized and used exclusively for research purposes.

## 3. Results

### 3.1. Sample Characteristics

Maternal, neonatal, and microscopic characteristics according to vaginal lactobacillary dominance are shown in Table 1. The two groups did not differ significantly in maternal age, pregestational BMI, gestational age at delivery, parity, education level, household income, newborn sex, birth weight, or birth length.

Smoking during pregnancy was more frequent among women with lactobacillary dominance than among those without dominance (11.8% vs. 1.7%, Fisher’s exact *p* = 0.027). Among neonatal anthropometric measures, head circumference differed significantly by lactobacillary dominance status (*p* = 0.026), whereas birth weight showed no significant difference (*p* = 0.090). Birth length did not differ between groups (*p* = 0.454).

Vaginal leukocyte density showed a borderline association (*p* = 0.073), while microscopic candidiasis was not significantly associated (*p* = 0.400). The composite vaginal microscopic indicator differed significantly according to *Lactobacillus* dominance status (*p* < 0.001). The Nugent score distribution showed minimal variability, with the majority of participants classified as normal flora (0–3: 97.9%), a small proportion as intermediate (4–6: 2.1%), and no cases of bacterial vaginosis (≥7).

### 3.2. Bivariate Associations with Neonatal Anthropometric Outcomes

Spearman correlation analyses showed no significant association between vaginal lactobacillary dominance and birth weight (ρ = 0.142, *p* = 0.090) or birth length (ρ = 0.063, *p* = 0.456). A weak but statistically significant positive correlation was observed with head circumference (ρ = 0.186, *p* = 0.025).

### 3.3. Linear Regression Analyses

In unadjusted linear regression models, vaginal lactobacillary dominance was not significantly associated with birth weight (B = 135.8 g, *p* = 0.067) or birth length (B = 0.22 cm, *p* = 0.469), but was associated with a higher head circumference (B = 0.52 cm, *p* = 0.035).

In adjusted models including gestational age at delivery, neonatal sex, pregestational BMI, and smoking during pregnancy, lactobacillary dominance was nominally associated with higher birth weight (B = 133.5 g, *p* = 0.043) and head circumference (B = 0.47 cm, *p* = 0.034), while no significant association was observed for birth length (B = 0.22 cm, *p* = 0.400). The corresponding standardized regression coefficients for lactobacillary dominance in the adjusted models were small in magnitude across outcomes (standardized β = 0.151 for birth weight, 0.062 for birth length, and 0.161 for head circumference), supporting the interpretation of limited relative effect size. After correction for the three primary anthropometric outcomes, the associations with birth weight and head circumference did not remain statistically significant. Bonferroni-adjusted *p*-values were 0.129 for birth weight, 1.000 for birth length, and 0.102 for head circumference. Benjamini–Hochberg false discovery rate-adjusted *p*-values were 0.065 for birth weight, 0.400 for birth length, and 0.065 for head circumference (Table 2).

### 3.4. Hierarchical Linear Regression Analyses

The incremental contributions of vaginal lactobacillary dominance (ΔR^2^ values) were small in magnitude across models. To evaluate whether vaginal lactobacillary dominance contributes additional explanatory value beyond established maternal and gestational determinants, hierarchical linear regression models were fitted for each neonatal anthropometric outcome (Table 3).

For birth weight, maternal factors (pregestational BMI and smoking) explained a negligible proportion of variance (R^2^ = 0.009), and the model was not statistically significant (*p* = 0.538). The addition of gestational age and neonatal sex substantially improved model fit (ΔR^2^ = 0.251). The inclusion of vaginal lactobacillary dominance further increased the explained variance (ΔR^2^ = 0.022). In the final adjusted model, lactobacillary dominance was associated with higher birth weight (B = 133.54 g, 95% CI 4.00 to 263.07, *p* = 0.043).

For birth length, maternal factors explained a small proportion of variance and the model was not statistically significant (R^2^ = 0.023, *p* = 0.200). Addition of gestational age and neonatal sex markedly improved model fit (ΔR^2^ = 0.273). Vaginal lactobacillary dominance did not contribute meaningful additional explanatory value (ΔR^2^ = 0.004) and was not significantly associated with birth length in the final model (B = 0.22 cm, 95% CI −0.30 to 0.74, *p* = 0.400).

For head circumference, maternal factors explained a small proportion of variance (R^2^ = 0.035), and the model was not statistically significant (*p* = 0.083). Addition of gestational age and neonatal sex significantly improved model fit (ΔR^2^ = 0.196). The inclusion of lactobacillary dominance further increased the explained variance (ΔR^2^ = 0.025). In the fully adjusted model, lactobacillary dominance remained associated with larger head circumference (B = 0.47 cm, 95% CI 0.04 to 0.91, *p* = 0.034).

Model diagnostics did not indicate major violations of linear regression assumptions. Standardized residuals were within ±3 in all three adjusted models. Cook’s distance values were below 1, with maximum values of 0.068 for birth weight, 0.074 for birth length, and 0.131 for head circumference. Maximum leverage was 0.206 in all three models. Collinearity was low across predictors, with variance inflation factors close to 1. Visual inspection of residual histograms, normal probability plots, and residual-versus-predicted plots did not suggest severe departures from normality or marked heteroscedasticity.

In extended hierarchical models additionally including maternal education and household income, maternal education was associated with neonatal head circumference (B = 0.52 cm, *p* = 0.029), whereas household income was not. Under this specification, the association between vaginal lactobacillary dominance and head circumference was attenuated and no longer reached statistical significance (*p* = 0.060).

For birth weight, the association with lactobacillary dominance was similarly attenuated (*p* = 0.061), and no additional associations were observed. For birth length, neither socioeconomic variables nor lactobacillary dominance were associated with the outcome. Inclusion of socioeconomic variables did not materially improve model fit across outcomes.

In sensitivity analyses additionally including maternal age and parity, vaginal lactobacillary dominance remained associated with birth weight (B = 151.9 g, *p* = 0.022) and head circumference (B = 0.53 cm, *p* = 0.018), while no association was observed for birth length (*p* = 0.315), consistent with the primary models.

In exploratory models, vaginal leukocyte density and microscopic candidiasis were not associated with neonatal anthropometric outcomes (all *p* > 0.100). In contrast, the composite vaginal microscopic indicator showed significant associations: intermediate status (vs balanced) was associated with lower birth weight (B = −183.2 g, *p* = 0.012) and smaller head circumference (B = −0.58 cm, *p* = 0.018), whereas no consistent associations were observed for birth length. No consistent gradient across categories was observed. These analyses were exploratory and were not adjusted for multiple comparisons.

## 4. Discussion

The principal finding is that lactobacillary dominance was not associated with neonatal anthropometric measures in unadjusted analyses, but showed modest associations with birth weight and head circumference in adjusted models. These findings should not be interpreted as evidence of an independent biological effect on fetal growth. The incremental contribution of lactobacillary dominance to explained variance was small (ΔR^2^ = 0.022 for birth weight and ΔR^2^ = 0.025 for head circumference), indicating limited clinical relevance. Similarly, the adjusted difference of approximately 133.5 g in birth weight should be interpreted as modest and insufficient, on its own, to support clinical predictive utility. In particular, the adjusted difference of approximately 0.47 cm in head circumference should be interpreted cautiously, because such a small magnitude may be influenced by routine anthropometric measurement variability. Overall, vaginal microscopic status is unlikely to represent a major determinant or clinically useful predictor of neonatal size.

The similarity between unadjusted and adjusted effect estimates suggests that the observed association was not materially driven by confounding in crude comparisons. Rather, the change in statistical significance appears to reflect increased precision after accounting for major determinants—particularly gestational age, fetal sex, and maternal characteristics—leading to narrower confidence intervals around a consistently modest effect size. This pattern is methodologically consistent with improved model specification rather than effect emergence, and supports a cautious interpretation: lactobacillary dominance does not exert a dominant effect on fetal growth, but may represent a secondary correlate within a broader network of maternal and gestational influences. The small incremental increases in explained variance (ΔR^2^ = 0.022 and 0.025, respectively) further reinforce that its contribution is marginal relative to established predictors such as gestational age, neonatal sex, pregestational BMI, and smoking during pregnancy. One unexpected finding was the higher proportion of smokers among women with lactobacillary dominance. This contrasts with reports linking tobacco exposure to variation in maternal and offspring microbiome profiles [14], although the small number of smokers in the present study (*n* = 11) limits interpretation. This imbalance may reflect sampling variability or residual confounding rather than a reproducible biological pattern. This finding should not be interpreted as suggesting that lactobacillary dominance counteracts or compensates for the established adverse effects of smoking on placental function and fetal growth. Because smoking was included in the adjusted models, its uneven distribution across lactobacillary dominance groups may also have contributed to some instability in the estimates, further supporting a cautious interpretation of the findings.

Existing literature supports the biological relevance of *Lactobacillus*-dominated vaginal ecosystems during pregnancy, particularly in relation to vaginal homeostasis, reduced dysbiosis, and adverse gestational outcomes such as preterm birth [3,4,5,8,16]. However, the present study did not assess species-level composition, microbial metabolites, epithelial barrier function, or inflammatory pathways. Therefore, the observed associations should be interpreted primarily as clinical-epidemiological signals derived from a microscopy-based marker, rather than as evidence of a defined mechanistic pathway linking vaginal lactobacillary dominance to fetal growth.

These results should also be interpreted cautiously in relation to sequencing-based literature. Molecular studies define vaginal microbial communities at the species or community-state level [1,2,6], whereas the present study used a semiquantitative Gram-stained microscopy marker and an operational threshold for lactobacillary predominance [8,16]. Consequently, the findings cannot be directly compared with community state type analyses or interpreted as evidence regarding species-level vaginal microbial composition. The restricted Nugent score variability and absence of bacterial vaginosis further indicate that the observed associations reflect variation within a predominantly normal microscopic profile rather than contrasts between normal flora and dysbiosis.

An important nuance relates to the inconsistent pattern of associations across neonatal anthropometric outcomes. A direct biological effect on fetal somatic growth would generally be expected to show a more coherent pattern across birth weight, birth length, and head circumference. In the present study, lactobacillary dominance was modestly associated with birth weight and head circumference in adjusted models, but not with birth length. This lack of association with linear growth limits the biological interpretation of the findings and argues against a uniform effect on fetal growth. Therefore, this pattern may reflect modest outcome-specific variability, residual confounding, measurement variability, or chance findings rather than a direct dimension-specific effect of microscopy-based lactobacillary dominance.

An alternative interpretation is that lactobacillary dominance may act less as a direct causal determinant and more as a marker of broader maternal physiological, behavioral and socioeconomic context. The attenuation of the associations after additional adjustment for education and household income suggests that lactobacillary dominance may partly reflect broader upstream determinants rather than an independent biological pathway affecting fetal growth. Socioeconomic position may influence maternal nutritional status, health literacy, hygiene practices, access to prenatal care, smoking patterns, sexual and reproductive health behaviors, and other unmeasured exposures that may shape both the vaginal ecological profile and neonatal growth. Evidence from large cohort analyses demonstrates that sociodemographic factors, particularly education, account for a substantial proportion of variation in vaginal microbiota structure during pregnancy [21]. Similarly, integrative frameworks emphasize that vaginal microbial composition is shaped by complex interactions between host, environmental, and behavioral factors rather than functioning as an isolated biological system [22]. Taken together, the modest and attenuated associations observed in the present study support the interpretation that lactobacillary dominance functions as a possible proxy indicator of a more favorable maternal health and socioeconomic environment, rather than as a primary biological driver of neonatal anthropometric variation. Because no formal mediation analysis was performed, these findings should be considered consistent with residual confounding or shared upstream pathways, not as evidence of a causal mediating mechanism.

An important consideration in interpreting these findings is the methodological approach. Most prior studies have relied on 16S rRNA sequencing and community state type classification [1,2,3,4,23], whereas the current analysis used routine Gram-stained microscopy and a pragmatic threshold for lactobacillary dominance. This difference limits direct comparability, as microscopy cannot resolve species-level variation or capture the full ecological complexity described in sequencing studies. This methodological contrast may partly explain the attenuated effect sizes and selective associations observed in the present study, and is consistent with sequencing-based studies that analyze neonatal anthropometry using gestational age- and sex-standardized z-scores, particularly in preterm cohorts with substantial variability in gestational age [24]; in contrast, the relatively homogeneous gestational age distribution in the present study and the explicit adjustment for gestational age in multivariable models support the use of absolute anthropometric measures.

The pattern observed across vaginal markers in this dataset further supports the specificity of lactobacillary dominance. Leukocyte density and microscopic candidiasis were not associated with neonatal anthropometric outcomes, while the composite vaginal microscopic indicator yielded only exploratory signals. This composite indicator is not a validated scoring system and should be interpreted as an exploratory proxy of vaginal ecological status. This distinction aligns with the conceptual separation between microbial composition and inflammatory activation [25], with lactobacillary dominance reflecting ecological structure [26], whereas leukocyte density reflects the host’s immune response [27], which may not directly translate into differences in fetal growth. Prior studies similarly emphasize that *Lactobacillus* dominance represents a central organizational feature of the vaginal ecosystem, while other markers capture distinct and less specific processes [1,2,22,28].

The absence of consistent associations for leukocyte density and microscopic candidiasis should also be interpreted in light of the fact that these markers capture different dimensions of vaginal microscopic status. Leukocyte density reflects a host inflammatory response, whereas candidiasis reflects fungal colonization, both of which may vary independently of lactobacillary predominance [15,16,17,18]. Accordingly, individual microscopic markers are unlikely to capture the full biological complexity potentially relevant to neonatal anthropometric variation.

Several implications arise from our findings. Microscopy-based lactobacillary dominance should not be interpreted as an independent determinant of neonatal anthropometry, but rather as one component within a multifactorial system. Clinically accessible markers such as lactobacillary dominance may retain some biological relevance, although their predictive value appears limited. These results also highlight the need for integrative approaches combining microbial, host, and environmental data to better understand the pathways linking maternal microbiota to perinatal outcomes.

The present findings do not support the use of vaginal lactobacillary dominance assessed by routine microscopy as a standalone predictor of neonatal anthropometric outcomes. In clinical practice, such an assessment may be more appropriately interpreted as a descriptive indicator of vaginal ecological status rather than as a tool for estimating neonatal size at birth [29]. Given its accessibility, microscopy-based evaluation may still be contextually relevant, particularly in settings where molecular profiling is unavailable [30]. Its interpretation should remain integrated with established maternal and gestational determinants, and its clinical utility is likely limited to complementing broader obstetric assessment. From a research perspective, these findings support the need for larger prospective studies that incorporate molecular characterization of the vaginal microbiota and longitudinal sampling throughout pregnancy [31,32]. Integrating microbial, maternal, and gestational data may help clarify the extent to which the microscopy-based lactobacillary dominance observed in this study reflects underlying vaginal ecological status or, to a limited degree, contributes to variation in neonatal anthropometric outcomes.

This study has several strengths. It used a prospective observational design with antenatal assessment of vaginal microscopic status before delivery, ensuring temporal separation between exposure measurement and neonatal outcomes. The study also examined routine, clinically available microscopy-based markers, addressing a question of direct relevance in settings where molecular microbiome profiling is not readily available. In addition, the analytical strategy included adjusted, hierarchical, sensitivity, and exploratory models, allowing a structured assessment of the consistency and specificity of the observed associations.

Several limitations should also be considered.

First, the sample size was modest, and the study was not specifically powered to detect small effect sizes or to support complex multivariable inference regarding fetal growth pathways. Although the adjusted models were deliberately parsimonious, the limited sample size increases the risk of imprecise estimates, residual confounding, and model instability. The observed associations should be interpreted as exploratory and hypothesis-generating, not as evidence of causal or clinically predictive relationships. At the same time, the possibility of type II error cannot be excluded, and the study may have had limited statistical power to detect small but potentially relevant associations, particularly after correction for multiple testing. Confirmation in larger, adequately powered, longitudinal cohorts is required.

Second, the study was conducted in a single center, which may limit generalizability to other populations and healthcare settings. The eligibility criteria should also be considered when interpreting the analytical sample; however, the macrosomia exclusion criterion did not affect the final sample or the reported associations, because no neonate had a birth weight ≥ 4000 g.

Third, vaginal microscopic status was assessed at a single time point in the third trimester and does not capture temporal variation in the vaginal ecosystem across pregnancy. This is particularly relevant because early pregnancy and mid-gestation represent critical windows for placental development, organogenesis, fetal programming, and the establishment of fetal growth trajectories. Therefore, a late-pregnancy vaginal smear should be interpreted as a marker of vaginal ecological status near delivery, rather than as a measure of microbial exposure throughout pregnancy. This limits temporal alignment between exposure and outcome and restricts any causal interpretation regarding fetal growth.

Fourth, routine microscopy provides a simplified representation of vaginal microbial ecology and cannot resolve species-level composition, strain-level differences, functional microbial activity, or overall microbial diversity. Therefore, this study should not be interpreted as a detailed microbiome analysis. The findings relate only to a clinically accessible microscopy-based marker of lactobacillary predominance and cannot distinguish potentially relevant *Lactobacillus* spp., such as *Lactobacillus crispatus* and *Lactobacillus iners* [16,30,33].

Although vaginal smears were evaluated within the routine laboratory workflow and internal quality-control procedures, the study did not include a formal study-specific inter-observer agreement analysis. Therefore, potential observer-dependent variability and exposure misclassification in semiquantitative microscopic classification cannot be fully excluded.

Fifth, although the main models were adjusted for key maternal and gestational factors, residual confounding cannot be excluded.

Sixth, some secondary and exploratory analyses, including extended models with socioeconomic variables and additional vaginal markers, were not part of the primary analytical framework and should be considered hypothesis-generating.

Although the three primary anthropometric outcomes were correlated, the nominal associations with birth weight and head circumference did not remain statistically significant after Bonferroni or Benjamini–Hochberg false discovery rate correction. This sensitivity analysis further indicates that the nominal findings are not robust to multiplicity correction and should not be interpreted as confirmatory evidence.

Finally, because the study focused on neonatal anthropometric outcomes at birth, the findings cannot be extrapolated to other perinatal outcomes not directly assessed in this analysis.

## 5. Conclusions

In this prospective cohort, vaginal lactobacillary dominance assessed by routine microscopy in late pregnancy showed modest associations with birth weight and head circumference after adjustment for key maternal and gestational factors, but no association with birth length. The magnitude of these associations, their small incremental contribution to explained variance, their attenuation after socioeconomic adjustment, and the timing of vaginal sampling all argue against interpreting lactobacillary dominance as an independent determinant of fetal growth.

Consistent with the observational design, these findings suggest that late-pregnancy lactobacillary dominance may represent a context-dependent marker of vaginal ecological status and broader maternal health or socioeconomic context, rather than a clinically useful predictor of neonatal anthropometry. Further studies with larger samples, longitudinal microbiome assessment, and formal evaluation of socioeconomic and behavioral pathways are needed to determine whether these associations are reproducible and have clinical relevance.

## Figures and Tables

**Table 1:** Maternal, neonatal, and microscopic characteristics according to vaginal lactobacillary dominance (*n* = 144).

Characteristic	*n* (%)/Median (IQR)			*p*
	Total Sample (*n* = 144)	Lactobacillary Dominance Absent (*n* = 59)	Lactobacillary Dominance Present (*n* = 85)	
Maternal				
Maternal age, years	30 (26–34)	30 (27–34)	29 (26–33)	0.166
Pregestational BMI, kg/m^2^	23.48 (21.09–27.18)	23.42 (21.22–26.40)	23.53 (21.09–27.55)	0.710
Gestational age at delivery, weeks	39 (38–39)	39 (38–39)	39 (38–39)	0.789
Parity (primiparous), *n* (%)	49 (34.0)	19 (32.2)	30 (35.3)	0.700
Smoking during pregnancy (yes), *n* (%)	11 (7.6)	1 (1.7)	10 (11.8)	0.027 ^1^
Education level, *n* (%)				0.241
≤12 years	77 (53.5)	35 (59.3)	42 (49.4)	
>12 years	67 (46.5)	24 (40.7)	43 (50.6)	
Household income category, *n* (%)				0.175
Low/very low	54 (37.5)	26 (44.1)	28 (32.9)	
Medium/high	90 (62.5)	33 (55.9)	57 (67.1)	
Neonatal				
Neonatal sex (male), *n* (%)	60 (41.7)	20 (33.9)	40 (47.1)	0.115
Birth weight, g	3230 (2955–3642)	3230 (2900–3450)	3250 (2985–3720)	0.090
Birth length, cm	50 (49–51)	50 (49–51)	50 (49–51)	0.454
Head circumference, cm	35 (34–36)	35 (33–35)	35 (34–36)	0.026
Microscopic vaginal profile				
Vaginal leukocyte density (high), *n* (%)	54 (37.5)	17 (28.8)	37 (43.5)	0.073
Microscopic candidiasis (present), *n* (%)	27 (18.8)	13 (22.0)	14 (16.5)	0.400

Data are presented as median (interquartile range, Q1–Q3) for continuous variables and *n* (%) for categorical variables. Continuous variables were compared using the Mann–Whitney U test. Categorical variables were compared using the chi-square test or Fisher’s exact test, as appropriate. ^1^ Fisher’s exact test.

**Table 2:** Bivariate and linear regression associations between vaginal lactobacillary dominance and neonatal anthropometric outcomes (*n* = 144).

Outcome	Vaginal Lactobacillary Dominance					
	Spearman ρ	*p*	Unadjusted B (95% CI)	*p*	Adjusted B (95% CI)	*p*
Birth weight, g	0.142	0.090	135.8 (−9.3 to 280.9)	0.067	133.5 (4.0 to 263.1)	0.043
Birth length, cm	0.063	0.456	0.22 (−0.38 to 0.81)	0.469	0.22 (−0.30 to 0.74)	0.400
Head circumference, cm	0.186	0.025	0.52 (0.04 to 0.99)	0.035	0.47 (0.04 to 0.91)	0.034

Data are presented as Spearman correlation coefficients (ρ) and unstandardized regression coefficients (B) with 95% confidence intervals. Unadjusted models include vaginal lactobacillary dominance as the sole independent variable. Adjusted models include gestational age at delivery, neonatal sex, pregestational BMI, and smoking during pregnancy. B coefficients represent the mean difference in the neonatal anthropometric outcome associated with lactobacillary dominance (present vs. absent).

**Table 3:** Hierarchical multiple linear regression models for birth weight (*n* = 144). Hierarchical multiple linear regression models for birth length (*n* = 144). Hierarchical multiple linear regression models for head circumference (*n* = 144).

Variable	Model 1 B (95% CI)	*p*	Model 2 B (95% CI)	*p*	Model 3 B (95% CI)	*p*
Pregestational BMI, kg/m^2^	1.15 (−14.14 to 16.45)	0.882	10.70 (−2.90 to 24.31)	0.122	10.11 (−3.36 to 23.57)	0.140
Smoking during pregnancy (yes vs. no)	−150.96 (−423.21 to 121.30)	0.275	−191.13 (−429.99 to 47.73)	0.116	−234.29 (−474.19 to 5.60)	0.056
Gestational age at delivery, weeks	—	—	175.07 (118.06 to 232.07)	<0.001	174.96 (118.59 to 231.33)	<0.001
Neonatal sex (male vs. female)	—	—	173.38 (44.12 to 302.65)	0.009	158.53 (29.89 to 287.17)	0.016
Vaginal lactobacillary dominance (present vs. absent)	—	—	—	—	133.54 (4.00 to 263.07)	0.043
Model fit	Model 1		Model 2		Model 3	
R^2^	0.009		0.260		0.281	
Adjusted R^2^	−0.005		0.239		0.255	
ΔR^2^ (vs previous)	—		0.251		0.022	
**Variable**	**Model 1** **B (95% CI)**	***p***	**Model 2** **B (95% CI)**	***p***	**Model 3** **B (95% CI)**	***p***
Pregestational BMI, kg/m^2^	−0.04 (−0.10 to 0.03)	0.264	0.01 (−0.05 to 0.06)	0.841	0.00 (−0.05 to 0.06)	0.869
Smoking during pregnancy (yes vs. no)	−0.82 (−1.91 to 0.28)	0.143	−0.98 (−1.93 to −0.04)	0.041	−1.06 (−2.02 to −0.10)	0.031
Gestational age at delivery, weeks	—	—	0.74 (0.52 to 0.97)	<0.001	0.74 (0.52 to 0.97)	<0.001
Neonatal sex (male vs. female)	—	—	0.72 (0.21 to 1.23)	0.006	0.70 (0.18 to 1.21)	0.008
Vaginal lactobacillary dominance (present vs. absent)	—	—	—	—	0.22 (−0.30 to 0.74)	0.400
Model fit	Model 1		Model 2		Model 3	
R^2^	0.023		0.295		0.299	
Adjusted R^2^	0.009		0.275		0.274	
ΔR^2^ (vs. previous)	—		0.273		0.004	
**Variable**	**Model 1** **B (95% CI)**	***p***	**Model 2** **B (95% CI)**	***p***	**Model 3** **B (95% CI)**	***p***
Pregestational BMI, kg/m^2^	0.05 (0.00 to 0.10)	0.047	0.08 (0.03 to 0.12)	0.001	0.07 (0.03 to 0.12)	0.001
Smoking during pregnancy (yes vs. no)	−0.41 (−1.29 to 0.48)	0.369	−0.58 (−1.39 to 0.22)	0.154	−0.74 (−1.54 to 0.07)	0.074
Gestational age at delivery, weeks	—	—	0.44 (0.24 to 0.63)	<0.001	0.44 (0.25 to 0.63)	<0.001
Neonatal sex (male vs. female)	—	—	0.77 (0.33 to 1.21)	0.001	0.72 (0.28 to 1.15)	0.001
Vaginal lactobacillary dominance (present vs. absent)	—	—	—	—	0.47 (0.04 to 0.91)	0.034
Model fit	Model 1		Model 2		Model 3	
R^2^	0.035		0.230		0.255	
Adjusted R^2^	0.021		0.208		0.228	
ΔR^2^ (vs. previous)	—		0.196		0.025	

Data are presented as unstandardized regression coefficients (B) with 95% confidence intervals. Model 1 includes pregestational BMI and smoking during pregnancy. Model 2 additionally includes gestational age at delivery and neonatal sex. Model 3 additionally includes vaginal lactobacillary dominance (present vs. absent). ΔR^2^ represents the increase in explained variance after inclusion of each block, based on F change statistics. All tests are two-sided; *p* < 0.05 was considered statistically significant.

## Data Availability

The datasets generated and/or analyzed during the current study are not publicly available due to ethical and data protection constraints related to participant confidentiality, but are available from the corresponding author on reasonable request and subject to institutional approval.

## Abbreviations

The following abbreviations are used in this manuscript:

BMI	Body mass index
CST	Community state type
CI	Confidence interval
ΔR^2^	Change in the coefficient of determination
IQR	Interquartile range
R^2^	Coefficient of determination
SD	Standard deviation
WHO	World Health Organization
